# Sensitizing properties of proteins: executive summary

**DOI:** 10.1186/2045-7022-4-10

**Published:** 2014-04-15

**Authors:** Lars K Poulsen, Gregory S Ladics, Scott McClain, Nancy G Doerrer, Ronald van Ree

**Affiliations:** 1 Allergy Clinic, Copenhagen University Hospital at Gentofte, Niels Andersens Vej 65, DK-2900 Hellerup, Denmark; 2 DuPont Pioneer Agricultural Biotechnology, DuPont Experimental Station, 200 Powder Mill Road, Wilmington, Delaware, USA; 3 Syngenta Crop Protection, LLC, 3054 E, Cornwallis Road, Research Triangle Park, North Carolina, USA; 4 ILSI Health and Environmental Sciences Institute, 1156 15th Street, NW, Suite 200, Washington DC 20005, USA; 5 Departments of Experimental Immunology and Otorhinolaryngology, Academic Medical Center, University of Amsterdam, Amsterdam 1066, GN, The Netherlands

**Keywords:** Allergic sensitization, Protein, Food allergy, Endogenous allergen

## Abstract

The scope of allergy risk is diverse considering the myriad ways in which protein allergenicity is affected by physiochemical characteristics of proteins. The complexity created by the matrices of foods and the variability of the human immune system add additional challenges to understanding the relationship between sensitization potential and allergy disease. To address these and other issues, an April 2012 international symposium was held in Prague, Czech Republic, to review and discuss the state-of-the-science of sensitizing properties of protein allergens. The symposium, organized by the Protein Allergenicity Technical Committee of the International Life Sciences Institute’s Health and Environmental Sciences Institute, featured presentations on current methods, test systems, research trends, and unanswered questions in the field of protein sensitization. A diverse group of over 70 interdisciplinary scientists from academia, government, and industry participated in the symposium. Experts provided overviews on known mechanisms by which proteins in food may cause sensitization, discussed experimental models to predict protein sensitizing potential, and explored whether such experimental techniques may be applicable in regulatory settings. Three accompanying reviews address critical factors and methods for assessing allergic sensitization: 1) food-and protein-related factors; 2) host-specific factors and 3) screening methods, *i.e.*, the ability of experimental models to predict the sensitizing potential of proteins and whether such models are applicable within regulatory settings.

## Introduction

The occurrence of food allergies is not evenly distributed globally and demonstrates some striking fluctuations:

• Although clinicians and immunologists have noted that good data from important parts of the world are lacking, there appear to be major regional differences in the prevalence of food allergy with variability in the pattern of foods to which patients react.

• Infants, older children, and adults react to different foods.

• There is some evidence that food allergy prevalence may be increasing, *i.e.*, a temporal change, in parallel to the rise in inhalation allergies seen in many parts of the world.

• Of the large variety of ingested food proteins, it is a relatively small number that seem to act as food allergens.

These paradoxes have been recognized for quite some time [1]; however, in recent years, new biological research has accumulated which may help explain these observations and use the knowledge to prevent or reduce the occurrence of new sensitizations and food allergies.

In evaluating the safety of new proteins in the human food chain, including the safety evaluation that precedes the introduction of genetically modified organisms (GMO), the predominant focus in relation to allergy has so far been on the elicitation phase of the allergic response. Fewer data and experimental models exist in relation to the mechanisms of induction of the sensitization phase or its prediction. To provide a state-of-the-science review, an international symposium titled “Sensitizing Properties of Proteins” was held in April 2012 in Prague, Czech Republic, bringing together over 70 scientists from academia, government, and industry. The meeting was organized by the Protein Allergenicity Technical Committee (PATC) of the International Life Sciences Institute’s (ILSI) Health and Environmental Sciences Institute (HESI). The purpose of the symposium was to present data on the current state of the science regarding the sensitizing properties of proteins in relation to food allergy. Experts from various disciplines and fields provided overviews on known mechanisms by which proteins in foods may cause sensitization. Also discussed was the ability of experimental models to predict the sensitizing potential of proteins and whether such experimental techniques are applicable within regulatory settings. Information and context from these presentations are described in three accompanying reviews [2-4]: 1) food-and protein-related factors; 2) host-immune factors and 3) screening methods, *i.e.*, the ability of experimental models to predict the sensitizing potential of proteins and whether such models are applicable within regulatory settings. This summary focuses on how the field of protein sensitization from food, including test systems, has developed, and identifies the most recent research trends and unanswered questions.

The process of sensitization to a food allergen represents an interplay between host-immune factors, the food, and the circumstances under which exposure takes place. It may be assumed that when infants develop food allergy to cow's milk protein after ingesting large amounts of milk the route of sensitization is oral via the digestive tract. On the other hand, it is well documented that a large proportion of food allergies occurring in adults and adolescents stems from cross-reactivity between food proteins and allergens derived from pollen where the route of sensitization is believed to be via inhalation. For foods such as peanuts and other legumes, fish, and shellfish, the route of sensitization is less clear with transdermal and inhalation routes having been suggested in addition to oral sensitization.

### Food and protein-related factors

Recent years have seen a dramatic increase in knowledge about food allergens, with detailed biochemical and biophysical characterization of the individual molecules [5]. The cloning and expression of purified protein allergens have allowed for more extensive studies. One important finding is the relative restriction of food allergens to a small number of protein families. While this is good news for using the existing knowledge base of allergens to predict and prevent potential cross-reactions when new proteins (genetically modified [GM] or conventional) are introduced into the food chain, it does not explain why these molecules become allergens. Developments in this field comprise studies of other biological capacities of the allergens, such as enzymatic (protease) activity and activation of the innate immune system, as well as broader approaches which focus on the whole food including matrix effects by lipids and cell walls. On the mechanistic level, an important factor is the immune system, where leukocytes can be modulated by enzymatic cleavage of receptors such as CD23 or CD25, or by activation of innate immune receptors such as Toll-like receptors by lipids or carbohydrates. Also the mucosal integrity may be at play, as some allergens with enzymatic activity have been shown to interfere with tight junctions and make the epithelium more susceptible to allergen uptake. Finally, protease-activated receptors on the epithelium may become stimulated initiating an inflammatory reaction. The way a food is processed may alter its interaction both with the digestive processes and the immune system with the gut microbiota as an intercalating factor. This is exemplified by stabilization of peanut allergens by roasting and the possible formation of new epitopes via Maillard reactions, which may take place when sugars bind to proteins under heat treatment.

While knowledge about allergens and triggers of the immune system has improved, the lectures and discussions during the symposium demonstrated that a clear hypothesis does not yet exist to explain the geographical and temporal variations in prevalence that food allergy epidemiologists observe. Clinically relevant cross-reactions may occur due to exposure to homologues of food allergens to which a patient has previously been sensitized, but apart from such reactions, it seems evident that a primary sensitization requires an exposure to the food in question. Having established this, however, there is little evidence of a dose–response relationship between the amount of ingested food and the risk of food allergy in individuals or in societies as a whole.

The epidemiological data, on the other hand, suggest that food allergies related to cross-reactive pollen allergens may be related to certain climate zones and specific fauna of a geographical region. Moreover, there may be some weak relationships to prevalent foods in an area, but the globalization of the food market tends to blur this effect.

See McClain et al. [2] for a more detailed discussion on the role of food- and protein-related factors in allergic sensitization.

### Host-immune factors

From a clinical perspective, it is crucial to identify risk factors in the individual. Previous diseases or co-morbidity are important in this respect, and it is essential to study the conditions by which an early IgE-sensitization to a food allergen develops into clinical food allergy. Allergic diseases have a strong genetic component, and recent findings that the filaggrin mutation may be a risk factor for the development of peanut allergy [6] marks a significant step toward understanding the full picture of the etiology of allergy. The apparent increase in food allergy prevalence during the last decades also calls for studies of the epigenetic regulation of the sensitization phase, a field which has not been studied so far in detail. Studies of elicitation of food allergies have addressed the importance of digestion and absorption processes, exercise, and drugs on these host-specific factors, but there is a strong need to extend these studies to include the sensitization phase.

Detailed knowledge about the epithelial barrier and antigen presenting cells, *i.e.*, dendritic cell subtypes that are specifically involved in the formation of Th2 cells and ultimately IgE-antibody production, may help with development of models that will predict allergenicity. In this respect, it is also pertinent to mention that quantitative knowledge of dose-sensitization relationships is extremely limited, but it is likely that both low- and high-dose tolerance induction may be relevant mechanisms for explaining the fact that just a small percentage of the population actually develops food allergy while the rest are tolerant to exposure to the same foods and protein allergens.

It was clear from symposium presentations that a multidisciplinary effort is necessary to delineate the pathway of allergic sensitization to a food allergen. Steps that must be considered are: 1) the passage of the allergen across the epithelium (in the airways or the digestive system) or the skin; 2) the antigen presenting cells, *i.e.*, dendritic cells; 3) the resulting meeting between dendritic cells and T cells; and finally, 4) the cooperation between the T-helper cell and the B cell.

See van Ree et al. [3] for a discussion of the complex interplay between the allergen and the host in allergic sensitization.

### Screening methods

Because knowledge of the processes leading to sensitization to food proteins is still quite fragmented, it is not surprising that there is no ideal model for risk evaluation of new proteins. In fact, a stable animal model or an *in vitro* system which will predict the sensitizing properties of a protein does not exist. However, there are interesting new developments in various research fields such as 1) *in silico* prediction of allergenic epitopes, 2) *in vitro* testing of stimulatory effects on the innate and the adaptive immune system, and 3) refined animal models where sensitization and reactions to clinical challenges act as outcome parameters. While these models may provide information about the mechanisms behind sensitization, they currently do not have the necessary robustness and credibility for regulatory purposes, nor can they predict allergy outcomes for a population. To fulfill such a scope, it would be necessary for models to provide an improved differentiation between some of today’s known food allergens and putative non-allergenic food proteins.

Several of the symposium presentations supported the notion that allergy and sensitization are in many ways different from more "classical" toxicology when it comes to test systems. First, as discussed above, the absence of an overwhelming theory of how sensitization takes place makes the approach of reductionism, known from other areas of biology and toxicology, difficult. That is, for example, when mutagenesis is believed to form an important step towards cancer, it makes sense to investigate for mutagenicity. But what are the crucial steps in sensitization? Other than identifying proteins that are closely biochemically related to known allergens, there is no clear consensus on risk parameters to characterize a new protein as a potential allergen.

Secondly, the very features of the immune system (*i.e.*, the IgE system is triggered by relatively low doses of antigen combined with mast cell activation which is one of the strongest physiological amplification systems) make quantitative modeling of dose–response extremely difficult and requires complex models. In this respect, the symposium's focus on sensitization was helpful, but also raised an important question about whether other important biomarkers exist beyond specific IgE on the way to becoming clinically allergic.

See Ladics et al. [4] for a discussion of screening methods and models that have been evaluated to predict protein sensitizing potential.

### Towards a unified understanding of food allergy development

In conclusion, while not establishing an overall mechanistic model of how food proteins may sensitize the human immune system, the symposium did establish some common challenges for predicting protein sensitization including (a) exposure routes; (b) frequency and dose of exposure; (c) dose–response relationships; (d) role of digestion, food processing, and the food matrix; (e) role of infection; (f) role of the gut microbiota; (g) influence of the structure and physicochemical properties of the protein; and (h) the genetic background and physiology of the consumers. The above models comprise a broad span from biochemical high-throughput models (phage-display technology or MHC-binding studies) over physiological models such as simulated gastro-duodenal digestion or synthetic organs to in vivo studies of allergenicity. The overall consensus of symposium participants was that screening models are extremely useful in the discovery and research phases of understanding the mechanisms of food allergy development. However, there are many methodological shortcomings and limitations identified with the current screening models (*e.g.*, the lack of a validated model that is predictive of protein allergenicity) which preclude their use for the safety assessment of novel proteins, new foods, and GM crops.

Since the World Health Organization (WHO)/Food and Agriculture Organization (FAO) sponsored one of the first comprehensive attempts to set up a total risk evaluation of GMO and allergenicity more than 10 years ago [7], many of the features of the original model have been refined. The discussion has now moved from looking only at cross-reactivity to known allergens to the increased expression of endogenous allergens and the main theme of the symposium, *i.e.*, the possibility of new proteins - unrelated to hitherto known allergens - becoming new allergens.

The 2012 symposium provided a forum to coalesce the state-of-the-science around predicting how and when food protein allergens initiate the allergic response. A generally increased interest in food allergy safety from the public has highlighted the need and benefits that will be gained once it is more fully understood how to predict when a protein may act as an allergen. As noted in existing regulatory safety guidance and numerous publications, allergy safety still relies on an accumulation of characterization studies rather than a single test (i.e., a weight-of-evidence approach).

## Competing interests

The authors declare that they have no competing interests.

## Authors’ contributions

LKP, GSL, SM, NGD, and RvR planned and organized the April 2012 Symposium on Sensitizing Properties of Proteins and contributed to this manuscript. All authors read and approved the final manuscript.
